# Case Report: Rare Case of *Staphylococcus pasteuri* Endocarditis

**DOI:** 10.1155/2023/4624492

**Published:** 2023-03-25

**Authors:** Esben Merrild, Mette Winther, Jonathan Nørtoft Dahl, Tine Sneibjerg Ebsen, Steffen Leth, Simon Winther

**Affiliations:** ^1^Department of Medicine, Randers Regional Hospital, Randers, Denmark; ^2^Department of Clinical Microbiology, Aarhus University Hospital, Aarhus, Denmark; ^3^Department of Cardiology, Gødstrup Regional Hospital, Herning, Denmark; ^4^Department of Clinical Medicine, Aarhus University, Aarhus, Denmark; ^5^Department of Infectious Diseases and Internal Medicine, Gødstrup Regional Hospital, Herning, Denmark

## Abstract

A 45-year-old woman was admitted with severe pain in the right leg and dyspnea. Her medical history included previous *Staphylococcus aureus* endocarditis, biological aortic valve replacement, and intravenous drug abuse. She was febrile but did not have any focal signs of infection. Blood tests showed raised infectious markers and troponin levels. Electrocardiogram showed sinus rhythm without signs of ischemia. Ultrasound revealed thrombosis of the right popliteal artery. The leg was not critically ischemic, and therefore, treatment with dalteparin was chosen. Transesophageal echocardiography showed an excrescence on the biological aortic valve. Empiric treatment for endocarditis was started with intravenous vancomycin, gentamicin, and oral rifampicin. Blood cultures subsequently grew *Staphylococcus pasteuri*. On day 2, treatment was changed to intravenous cloxacillin. Due to the comorbidity, the patient was not a candidate for the surgical treatment. On day 10, the patient developed moderate expressive aphasia and weakness in the right upper limb. Magnetic resonance imaging showed micro-embolic lesions scattered across both hemispheres of the brain. Treatment was changed from cloxacillin to cefuroxime. On day 42, infectious markers were normal, and echocardiography showed regression of the excrescence. Antibiotic treatment was stopped. Follow-up on day 52 did not show any signs of active infection. However, on day 143, the patient was readmitted with cardiogenic shock due to aortic root fistulation to the left atrium. She quickly deteriorated and died.

## 1. Introduction

Infective endocarditis (IE) is a potential deadly disease requiring early recognition and treatment. Prosthetic valve endocarditis (PVE) accounts for 10–30% of all cases of IE. Early PVE is defined as endocarditis within 1 year of surgery and is often due to coagulase-negative staphylococci (CoNS) [1, 2].

CoNS have historically been regarded as a low-virulent group of pathogens. However, previously, subspecies have seldom been identified. Therefore, the virulence of certain subspecies might be underreported [3].

*Staphylococcus pasteuri* is a Gram-positive CoNS previously isolated from food products and known as commensals of the human skin microbiota. However, its natural habitat is poorly understood [4].

*S. pasteuri* is a rare cause of IE and to the best of our knowledge, only one such case has previously been described [5]. We present the first case of *S. pasteuri* PVE.

## 2. Case Presentation

**Figure 1:**
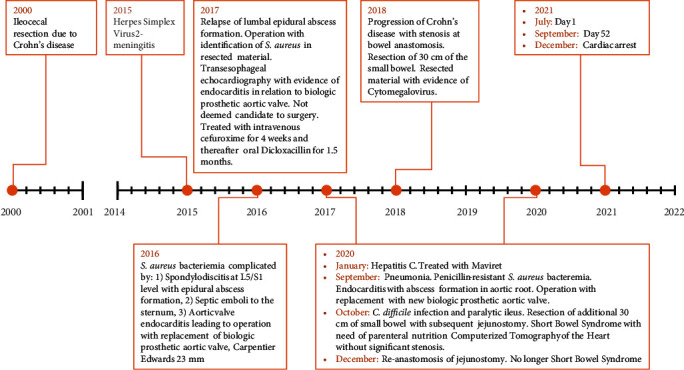
Timeline (see text for further details).

A 45-year-old woman was admitted with sudden onset of severe pain in the right leg and dyspnea. The pain was localized to the right popliteal region and described as tightness/heaviness with exacerbation on mobilization. In the two prior months, the patient had been experiencing chest pain and progressively worsening dyspnea limiting physical activity to a walking distance of 50 m. The chest pain was sharp, left-sided, and pleuritic with exacerbation on inspiration. Her medical history included three episodes of endocarditis with *Staphylococcus aureus* treated twice with biological aortic valve replacement, and the latest aortic valve replacement was performed 267 days prior to admission. Additionally, her past medical history included chronic hepatitis-C-infection and Crohn's disease complicated by ileocecal resection and previous intravenous drug abuse (Figure 1).

On examination, the patient was febrile with a temperature of 38.4°C, but otherwise with normal vital parameters. Physical examinations revealed severe tenderness at the right popliteal region without swelling or apparent signs of trauma or infection and with weak pedal pulses. There were no obvious signs of an infectious focus. Cardiovascular examination was with normal heart and lung auscultation and with no signs of peripheral edema. Electrocardiogram showed sinus rhythm without any other abnormalities.

Blood tests showed C-reactive protein level of 202 mg/L [<0.8 mg/L] with neutrophils of 9.15 × 10^9^/L [2.0 × 10^9^/L; 7.0 × 10^9^/L], hemoglobin of 6.1 mmol/L [7.3; 9.5 mmol/L], D-dimer of 2.1 mg/L [<0.5 mg/L], and two sets of troponin I, which were 4 hours apart with values of 3,051 ng/L and 3,111 ng/L [<24 ng/L], respectively.

**Figure 2:**
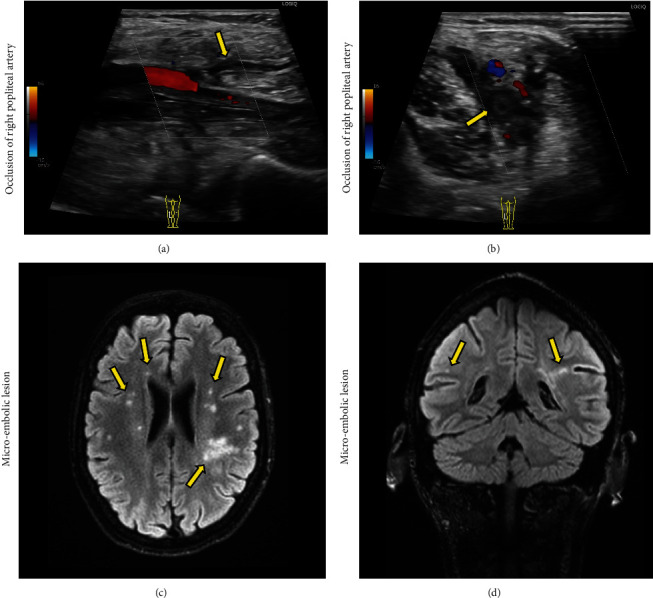
Ultrasound images of the right popliteal artery in the long and short axis (a and b, respectively) show partial obstruction of flow as evident on color doppler imaging (yellow arrows). Magnetic resonance imaging in the horizontal and coronal planes (c and d, respectively) shows multiple micro-embolic lesions scattered across both hemispheres of the brain (yellow arrows).

Thrombosis with partial obstruction of the right popliteal artery was diagnosed by ultrasound of the right lower extremity (Figure 2). A subsequent computed tomography (CT) confirmed this finding. The leg was not found to be critically ischemic, and conservative treatment with dalteparin 200 IE/kg was initiated.

**Figure 3:**
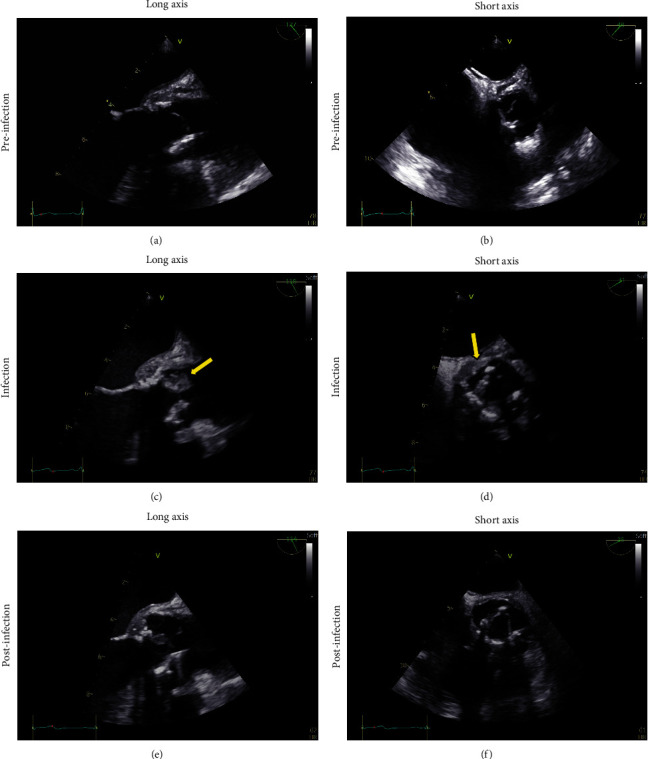
Transesophageal echocardiography images of the biological aortic valve prosthesis seen before (a and b), during (c and d), and after (e and f) active infection. Images are shown in long axis (a, c, and e) and short axis (b, d, and f). Yellow arrows point to the excrescence on the non-coronary cusp of the biological aortic valve prosthesis.

Transthoracic echocardiography (TTE) showed an ejection fraction of 60% with no regional wall abnormalities. The biological aortic valve prosthesis was found to be in situ and without paravalvular leakage; however, an 8 × 9 mm excrescence was found on the non-coronary cusp. Transesophageal echocardiography (TEE) confirmed this finding (Figure 3).

Treatment for endocarditis was initiated on an empiric basis with intravenous administration of vancomycin 1,000 mg twice daily, gentamicin 270 mg once daily, and oral rifampicin 600 mg twice daily according to national recommendations [6]. Prior to treatment initiation, two sets of blood cultures had been drawn. *S. pasteuri* grew in four out of four samples and was susceptible to cloxacillin and rifampicin. Hence, treatment was changed on day 2 to intravenous cloxacillin 3,000 mg four times daily in combination with oral rifampicin 600 mg twice daily according to national recommendations [6]. Due to comorbidity, the patient was not a candidate for surgical treatment.

Ten days after admittance, the patient developed several episodes of expressive aphasia and weakness in the right upper extremity. Magnetic resonance imaging (MRI) of the brain was performed showing ischemic changes in the left frontal lobe with a multi-focal distribution suggestive of micro-embolic events (Figure 2). Treatment was changed from cloxacillin 3 g four times daily to intravenous cefuroxime 3 g three times daily due to improved penetration through the blood–cerebrospinal fluid/blood–brain barrier into the central nervous system (CNS). Treatment with rifampicin was continued.

Subsequent MRI of the brain on day 32 showed no signs of progression of the ischemic changes, including no signs of abscess formation.

During the following days, the patient began to demonstrate low compliance. Contributing factors were the development of red urine, a common side effect of rifampicin, along with the high frequency of antibiotic administration. At this stage, the patient had been afebrile for 10 days and with good clinical response with a C-reactive protein (CRP) of 68 mg/L and neutrophils of 7.6 × 10^9^. Thus, on day 33, the attending physicians decided to stop treatment with rifampicin and continued with intravenous cefuroxime thrice daily.

On day 40, TEE showed regression of the excrescences. However, a remaining mobile, pendulating elongated element was found on the non-coronary cusp measuring 1 × 6 mm. A small transvalvular insufficiency was found, but no signs of paravalvular leakage. Additionally, mobile elements were found on the anterior and posterior leaflets of the mitral valve measuring 2 × 3 mm each. Blood tests showed normalization of infection parameters. The plan for a total of 6 weeks of intravenous antibiotics was maintained, as both cardiac and cerebral imaging showed good responses with concurrent normalization of both clinical and biochemical findings.

On day 42, the patient was discharged after a complete 42-day course of antibiotic treatment.

Follow-up at the outpatient clinic on day 52 showed no signs of active infection including normal blood cultures and blood samples with normal infection parameters. A subsequent TTE was planned; however, the patient never showed up for this.

On day 143, the patient developed acute onset of dyspnea. Upon arrival to the emergency department, the patient had developed cardiac arrest with asystole, and she was successfully resuscitated and transferred to intensive care unit with extreme hypotension. TEE revealed a pendulating excrescence on the aortic valve; furthermore, fistulation with shunting of blood from the aortic root into the left atrium was seen. Acute rescue thoracic surgery was available but not deemed a viable option. The patient deteriorated and supportive treatment was stopped. The patient died from circulatory collapse on the day of admission. Blood cultures subsequently revealed four out of four samples with S. *aureus* and no evidence of *S. pasteuri*.

## 3. Microbial Assessment

**Figure 4:**
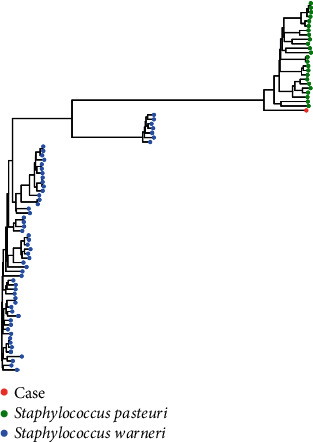
K-mer tree.

By use of Matrix-assisted laser desorption/ionization-time of flight (MALDI-TOF) mass-spectrometry (MALDI Biotyper, Bruker Daltonics, Bremen, Germany), the bacterium in this patient was identified as *S. pasteuri* (log-score 1.9–2.26). In order to confirm the strain to species level, we performed whole-gene-sequencing of the isolate with the Illumina Nova Seq System producing 2 × 150 bp paired-end reads by using Twist-library. Reads were assembled using Unicycler v. 0.4.4 to eight contigs with a total sequence length of 2.468.945 bp with a G + C content of 31.43%. Finally, we generated a simple K-mer by pooling genomes of *S. pasteuri* and *Staphylococcus warneri* in the Genome Taxonomy Database (see Figure 4 and supplementary for details).

## 4. Discussion

Here we described a rare case of significant *S. pasteuri* prosthetic aortic valve endocarditis in a 45-year-old-woman. To the best of our knowledge, this is the first case of *S. pasteuri* PVE. From the literature review, only one case of endocarditis with *S. pasteuri* has been described, and this was in a patient with aortic valve disease and previous intravenous drug abuse like in our patient [5].

Only one other case [7] has described *S. pasteuri* bacteremia, and this was in a 75-year-old woman with leukemia and fever. In the remaining cases the bacterium was isolated from the urethra of an immunocompromised patient [8] and from the thumb of a patient who developed osteomyelitis after accidental injection with cannabidiol oil [9].

As of December 2017, the genus Staphylococcus consists of 49 validly described species including 25 subspecies [10]. *S. pasteuri* is known to be closely related to *S. warneri.* Thus, correct identification of *S. pasteuri* may be challenging. In the present case, the identification of *S. pasteuri* was rigorously performed with MALDI-TOF mass-spectrometry and confirmed by whole-gene-sequencing.

CoNS are a heterogenous group, historically differentiated as less pathogenic with infections more slowly progressing than that of the coagulase positive *S. aureus*. CoNS possess fewer virulence properties than *S. aureus.* Nonetheless, due to changes in procedures and host factors, and more cases of having prosthetic valves, the bacteria have become a major nosocomial pathogen [3].

In a study using Danish nationwide registries from 2010 to 2017, overall in-hospital mortality for IE was 18.7%, whereas the mortality was 16.6% when CoNS were the cause. CoNS were the cause of IE in 6.2% of all cases. However, among patients with prosthetic heart valves, CoNS were the most common causative pathogen attributing to 31.8% of PVEs [11].

In the described case, several possible explanations for the infection exist. Nosocomial transmission could have happened during surgery with aortic valve replacement or in relation to re-anastomosis of the small bowel. The fact that many CoNS colonize and spread through implanted foreign bodies, e.g., indwelling intravenous catheters support this hypothesis [3]. Another explanation is community-acquired transmission that can occur [5].

According to Danish national guidelines for management of methicillin susceptible staphylococci, the patient was initially treated with intravenous cloxacillin 3,000 mg four times daily in combination with oral rifampicin 600 mg twice daily due to the existence of the prosthetic valve [6]. After the micro-embolic complication of the brain, treatment was changed to cefuroxime 3,000 mg three times daily and oral rifampicin 600 mg twice daily for improved drug penetration to the CNS, which is Danish practice. Total antibiotic treatment was scheduled for 42 days; however, rifampicin was discontinued on day 33, i.e., 9 days before schedule.

An interesting observation is the fact that the patient developed embolisms to the brain despite concurrent dalteparin and antibiotic treatment. A previous study by Lee et al. [12] showed that the role of anticoagulant therapy in prevention of embolisms was limited in patients with IE undergoing antibiotic therapy. Likewise, a randomized trial with aspirin treatment in patients with IE showed no reduction in embolic events and a trend toward higher incidence of bleeding [13]. The indication for dalteparin use in our case was treatment of arterial embolism of the leg. However, the development of embolisms to the brain, despite concurrent dalteparin, adds to the notion that primary prophylactic anticoagulant therapy may not be warranted in patients with IE undergoing antibiotic therapy.

*S. aureus* was found in relation to the final, fatal admission, after out-patient follow-up and after end of antibiotic treatment—indicative of new infection. Hence, it is reasonable to believe that *S. pasteuri* PVE was successfully treated conservatively with 6 weeks of antibiotics.

## 5. Conclusion

*S. pasteuri* is a rare cause of endocarditis. *S. pasteuri* is a CoNS, a group of bacteria that are generally regarded as low virulence. However, when foreign bodies are involved, the risk of significant disease increases.

In this case, the patient was diagnosed with *S. pasteuri* PVE complicated by embolic phenomena to both the leg and the brain. Antibiotic treatment resulted in a good clinical response; however, the patient later died due to *S. aureus* PVE. Increased identification of species within the CoNS group may lead to better understanding of differing pathogenicity and treatment strategies.

## Data Availability

The assembled genome can be found at National Library of Medicine under GenBank access ID JAMXHQ000000000.1.

## Supplementary Materials

Supplementary Materials
**Video** Transesophageal echocardiography images during infection show a pendulating excrescence on the non-coronary cusp of the biological aortic valve prosthesis. Images shown are in the long and short axis, respectively.

## References

[1] Habib G., Lancellotti P., Antunes M. J., Bongiorni M. G., Casalta J. P., Del Zotti F. (2015). 2015 ESC guidelines for the management of infective endocarditis: the task force for the management of infective endocarditis of the European Society of Cardiology (ESC). Endorsed by: European Association for Cardio-Thoracic Surgery (EACTS), the European Association of Nuclear Medicine (EANM). *European Heart Journal*.

[2] Hill E. E., Herijgers P., Herregods M. C., Peetermans W. E. (2006). Evolving trends in infective endocarditis. *Clinical Microbiology and Infection*.

[3] Becker K., Heilmann C., Peters G. (2014). Coagulase-negative staphylococci. *Clinical Microbiology Reviews*.

[4] Savini V., Catavitello C., Bianco A., Balbinot A., D’Antonio D. (2009). Epidemiology, pathogenicity and emerging resistances in Staphylococcus pasteuri: from mammals and lampreys, to man. *Recent Patents on Anti-Infective Drug Discovery*.

[5] Ramnarain J., Yoon J., Runnegar N. (2019). Staphylococcus pasteuri infective endocarditis: a case report. *IDCases*.

[6] https://www.cardio.dk/appendix-7-1.

[7] Savini V., Catavitello C., Carlino D. (2009). Staphylococcus pasteuri bacteraemia in a patient with leukaemia. *Journal of Clinical Pathology*.

[8] Morfin-Otero R., Martínez-Vázquez M. A., López D., Rodríguez-Noriega E., Garza-González E. (2012). Isolation of rare coagulase-negative isolates in immunocompromised patients: Staphylococcus gallinarum, Staphylococcus pettenkoferi and Staphylococcus pasteuri. *Annals of Clinical and Laboratory Science*.

[9] Santoiemma P. P., Kalainov D. M., Mehta M. P., Bolon M. K. (2020). An unusual case of Staphylococcus pasteuri osteomyelitis. *Infectious Disease Reports*.

[10] Carroll K. C., Pfaller M. A., Landry M. L. (2019). *Manual of Clinical Microbiology*.

[11] Østergaard L., Voldstedlund M., Bruun N. E. (2022). Temporal changes, patient characteristics, and mortality, according to microbiological cause of infective endocarditis: a nationwide study. *Journal of the American Heart Association*.

[12] Lee S.-J., Oh S.-S., Lim D.-S., Hong S.-K., Choi R.-K., Park J.-S. (2014). Usefulness of anticoagulant therapy in the prevention of embolic complications in patients with acute infective endocarditis. *BioMed Research International*.

[13] Chan K. L., Dumesnil J. G., Cujec B. (2003). A randomized trial of aspirin on the risk of embolic events in patients with infective endocarditis. *Journal of the American College of Cardiology*.

