# Supplementary material for: Case Report: Rare Case of Staphylococcus pasteuri Endocarditis
Source: Case Rep Cardiol. 2023 Mar 25;2023:4624492. doi: 10.1155/2023/4624492 (PMC10066806; doi:10.1155/2023/4624492)
Supplement: Supplementary Materials — Video Transesophageal echocardiography images during infection show a pendulating excrescence on the non-coronary cusp of the biological aortic valve prosthesis. Images shown are in the long and short axis, respectively. [file 4624492.f1.docx]

# Supplementary : PLEASE NOTE THAT THE SUPPLEMENTARY FILES ARE UPLOADED UNDER FIGURES & TABLES SECTION.

## Video

Transesophageal echocardiography images during infection show a pendulating excrescence on the noncoronary cusp of the biological aortic valve prothesis. Images shown are in the long and short axis, respectively.
